# Disease Severity and Progression in Progressive Supranuclear Palsy and Multiple System Atrophy: Validation of the NNIPPS – PARKINSON PLUS SCALE

**DOI:** 10.1371/journal.pone.0022293

**Published:** 2011-08-04

**Authors:** Christine A. M. Payan, François Viallet, Bernhard G. Landwehrmeyer, Anne-Marie Bonnet, Michel Borg, Franck Durif, Lucette Lacomblez, Frédéric Bloch, Marc Verny, Jacques Fermanian, Yves Agid, Albert C. Ludolph, Peter N. Leigh, Gilbert Bensimon, on behalf of the NNIPPS Study Group

**Affiliations:** 1 Département de Pharmacologie Clinique, Hôpital de la Pitié-Salpêtrière, APHP, UPMC Pharmacologie, Paris 6, UMR 7211, Paris, France; 2 Service de Neurologie, Centre Hospitalier du pays d'Aix, Aix en Provence, France; 3 Department of Neurology, University of Ulm, Ulm, Germany; 4 Fédération de Neurologie, INSERM, Centre d'Investigation Clinique CIC9503, Hôpital de la Pitié-Salpêtrière, APHP & UPMC Université Paris 6, Paris, France; 5 Service de Neurologie, Hôpital Pasteur, Nice, France; 6 Service de Neurologie, Hôpital Gabriel Montpied, Clermont-Ferrand, France; 7 Département de Neurologie, Hôpital de la Pitié-Salpêtrière, APHP, UPMC, Pharmacologie, Paris 6, INSERM, UMR-S 678, Paris, France; 8 Centre de Gériatrie, Groupe Hospitalier Pitié-Salpêtrière & UPMC Université Paris 6, Assistance Publique-Hôpitaux de Paris, Paris, France; 9 Service de Biostatistiques, Hôpital Necker-Enfants Malades, APHP, Paris, France; 10 Clinical Neurosciences, Brighton and Sussex Medical School, Trafford Centre for Biomedical Research, University of Sussex, Falmer, United Kingdom; University of California Los Angeles, United States of America

## Abstract

**Background:**

The Natural History and Neuroprotection in Parkinson Plus Syndromes (NNIPPS) study was a large phase III randomized placebo-controlled trial of riluzole in Progressive Supranuclear Palsy (PSP, n = 362) and Multiple System Atrophy (MSA, n = 398). To assess disease severity and progression, we constructed and validated a new clinical rating scale as an ancillary study.

**Methods and Findings:**

Patients were assessed at entry and 6-montly for up to 3 years. Evaluation of the scale's psychometric properties included reliability (n = 116), validity (n = 760), and responsiveness (n = 642). Among the 85 items of the initial scale, factor analysis revealed 83 items contributing to 15 clinically relevant dimensions, including *Activity of daily Living/Mobility*, *Axial bradykinesia*, *Limb bradykinesia*, *Rigidity*, *Oculomotor*, *Cerebellar*, *Bulbar/Pseudo-bulbar*, *Mental*, *Orthostatic*, *Urinary*, *Limb dystonia*, *Axial dystonia*, *Pyramidal*, *Myoclonus* and *Tremor*. All but the *Pyramidal* dimension demonstrated good internal consistency (Cronbach α≥0.70). Inter-rater reliability was high for the total score (Intra-class coefficient = 0.94) and 9 dimensions (Intra-class coefficient = 0.80–0.93), and moderate (Intra-class coefficient = 0.54–0.77) for 6. Correlations of the total score with other clinical measures of severity were good (rho≥0.70). The total score was significantly and linearly related to survival (p<0.0001). Responsiveness expressed as the Standardized Response Mean was high for the total score slope of change (SRM = 1.10), though higher in PSP (SRM = 1.25) than in MSA (SRM = 1.0), indicating a more rapid progression of PSP. The slope of change was constant with increasing disease severity demonstrating good linearity of the scale throughout disease stages. Although MSA and PSP differed quantitatively on the total score at entry and on rate of progression, the relative contribution of clinical dimensions to overall severity and progression was similar.

**Conclusions:**

The NNIPPS-PPS has suitable validity, is reliable and sensitive, and therefore is appropriate for use in clinical studies with PSP or MSA.

**Trial Registration:**

ClinicalTrials.gov NCT00211224

## Introduction

Progressive Supranuclear Palsy (PSP) and Multiple System Atrophy (MSA), sometimes termed ‘parkinson plus’ syndromes, account for 10–20% of parkinsonian syndromes [1]–[3], although these figures may be an overestimate being derived from autopsy studies. Both diseases are associated with severe disability and early death [4]–[7]. PSP and MSA most commonly present with an akinetic-rigid syndrome, with additional features such as dysautonomia and cerebellar features in MSA, or oculomotor, bulbar, cognitive and behavioral abnormalities in PSP [2], [8]. However, the expression of these features is variable during the evolution of these syndromes, and although some are regarded as typical of PSP (e.g., supranuclear ophthalmoplegia, dementia) or of MSA (e.g., dysautonomia, cerebellar syndrome), there is considerable overlap between the two disorders [8]–[12]. In addition, if we are to study these disorders early in their evolution, then a generic ‘parkinson plus’ scale is required, and such a scale should capture all important aspects of the severity of the clinical syndromes. To date, no scale designed to assess severity and disease progression over the many functional dimensions relevant to parkinson plus syndromes has been developed and fully validated. Although the Unified Parkinson's Disability Rating Scale (UPDRS) [13] has been used in PSP [11], [14] and MSA [15], [16], assessment of its metric qualities has not been completed in this population. While the PSP Rating Scale (PSP-RS) [5],[17] and the Unified Multiple System Atrophy Rating Scale (UMSARS) [18]–[20] were designed specifically for PSP and MSA respectively, neither of these scales was designed to cover the full spectrum of disability in atypical parkinsonian (‘parkinson plus’) syndromes or to capture functional deficits in early MSA or PSP when the diagnosis remains uncertain. Indeed, a scale that can with equal validity be applied to either disease in the early stages is as important in the investigation of natural history as it is in clinical trials. As part of the NNIPPS study [8] we therefore developed a clinical scale applicable in large multicentre trials that would allow evaluation of atypical parkinsonian syndromes at all stages, while also providing useful measures of change across the whole course of disease evolution.

Thus our main objectives were to evaluate disease severity and progression in PSP and MSA in relation to treatment; to ascertain that prognostic factors at entry were balanced between treatment groups; and to provide candidate covariates for survival analysis. Critically, the NNIPPS study was designed with stratification according to diagnosis at entry (PSP versus MSA) and required balanced numbers of patients in each stratum. This allows independent assessments of the results for each condition, and unbiased comparisons of symptom severity between diseases. Here we present the symptom severity profile and rate of progression in each disorder as evaluated with the NNIPPS-PPS scale, along with its psychometric properties, including face and content validity, construct validity, inter-rater reliability, and responsiveness.

## Materials and Methods

### Ethics approval

The protocol and amendments were reviewed and approved by the Comité de Protection des Personnes of Pitié-Salpêtrière Hospital (France), the UK Multicentre Research Ethics Committee (MREC), (UK), Ethikkommission of the University of Ulm, (Germany), and by local Institutional Review Boards (Ethics Committees) where appropriate (UK, Germany).

### Trial design

The NNIPPS study was granted approval by the relevant Institutional review boards and all subjects gave fully informed signed consent before enrolment. Patients with an akinetic-rigid syndrome diagnosed as PSP or MSA according to the NNIPPS diagnostic criteria [8] were eligible. Details of the therapeutic trial design and results have been reported previously [8]. In brief, the intent to treat population comprised 760 patients (362 PSP and 398 MSA) recruited in 44 centers in the UK, France and Germany. Patients were stratified according to diagnosis and randomized double-blind to riluzole or placebo. The study was powered to demonstrate efficacy within each strata independently. The primary efficacy measure was survival, and secondary endpoints were rates of change in functional scores. Patients were evaluated 6-monthly for 3 years until death or the administrative cut-off date.

### Scale construction

Prior to the start of the trial, items were selected through expert consensus as part of a broad clinical description of both MSA and PSP. The dimensions included (i) functional disability (activities of daily living), (ii) mental function (cognition, mood & behavior); (iii) extra-pyramidal motor disability (rigidity, bradykinesia), (iv) tremor, (v) oculomotor function, (vi) cerebellar signs, (vii) pyramidal signs, (viii) dysautonomia, (ix) bulbar/pseudobulbar symptoms, (x) myoclonus, and (xi) dystonia. Items were selected from the following scales available at that time, the UPDRS (all items from Mental, ADL and Motor examination sections) [13], the PSP-RS (six items from the mental section) [17], three items from the International Cooperative Ataxia Rating Scale (ICARS) [21], the global ataxia score of the Expanded Disability Status Scale (EDSS ) [22], and four items evaluating orthostatic signs and three for urinary signs from the Autonomic Symptom Profile [23] adapted to interview record instead of self-rating. Additional items were included to assess *oculomotor signs*, *dystonia*, *myoclonus*, *pyramidal signs*, *sitting down* and *strength of cough*.

A preliminary version of 109 items was evaluated in a pilot study to check each item and category wording. Redundant or inappropriate items were eliminated to obtain the first version comprising 85 items to be tested. Severity levels of items ranged from 0 (“normal”) to a maximum of 6 (very severe), with a majority of items (65) scored on a 5-point scale (0–4) (Supporting information S1). Four sections were interview based with patient and/or caregiver (Mental, Activities of Daily Living-ADL, orthostatic and urinary signs), eleven were assessed through examination. Time to complete the scale was 30–45 minutes depending on clinical state of patient. Throughout the study, the scale was completed in all centres using an English version.

### Psychometric properties

According to the recommendations of the American Psychological Association [24], we evaluated face and content validity, construct validity (Factor analysis, internal consistency, convergent and predictive validity) [25] (Supporting information S2). Total score and dimensional sub scores were obtained from summing item scores overall or within dimensions, respectively.

For convergent validity we used Spearman rank correlations with other clinical measures *a priori* considered as related to disease severity. These included the Hoehn & Yahr staging (HYS) [26], Schwab & England Activity daily Living scale (SEADL) [27], the Mini Mental State Examination (MMSE) [28], the Frontal Assessment Battery (FAB) [29], the Clinician Global Impression of disease severity (CGI-ds) [25], 6 visual analog scales (VAS) of syndromes severity (akinesia-rigidity, dysautonomia, cerebellar, pyramidal, bulbar/pseudo-bulbar, behavioral and cognitive dysfunction), a CGI–dysautonomia score [8] and two quality of life scales, the Parkinson's disease questionnaire (PDQ-8) [30] and the short form 36 health questionnaire (SF36) [31]. Correlations were considered for rho≥0.40. For predictive validity, relation between scores at inclusion and survival was evaluated using univariate and multivariate Cox model analysis [32].

### Reliability

An inter-rater reliability study was conducted with sub-samples of patients recruited from 11 centers (France: n = 3, UK: n = 3, Germany: n = 5). At inclusion, patients were evaluated twice independently on the same day. To assess inter-rater agreement, Cohen's linear weighted kappa (κ_w_) or simple kappa (κ) for binary items was calculated for each item [33], [34]. For the dimensional sub scores and the total score, Fisher's intra-class coefficients (ICC) were computed using analysis of variance (ANOVA) with a one-way random effect model [35]. Inter-rater reliability coefficients were interpreted according to proposed standards for strength of agreement as: ≤0.20 = poor, 0.01–0.20 = slight, 0.21–0.40 = fair, 0.41 to 0.60 = moderate, 0.61–0.80 = substantial, and 0.81–1.0 = almost perfect [36]. Individual item strength of agreement was considered as acceptable for κ>0.40 (moderate to almost perfect); for dimensional sub scores, ICC threshold for acceptability was raised to 0.70. Internal consistency of the total and dimensional scores was evaluated through Cronbach α coefficients and considered acceptable for α>0.70.

### Sensitivity to change

For each patient with at least two usable assessments, repeated measurements of the NNIPPS-PPS total score and dimensional sub scores were summarized by the slope of change (annual rate of change in scores), using unweighted least-square regression estimates [37]. To assess independence of change relative to severity stage, we compared total score slope of change across the whole range of severity grades defined by the CGI-ds (one-way anova with test of trend). To test scale sensitivity to treatment effects, mean slopes were compared between the treated and placebo groups using two-way anova including treatment, diagnostic strata, and treatment by strata interaction factors.

Responsiveness was further evaluated using effect size (ES) defined as the ratio of the difference in slopes of change between treatment groups to the Standard Deviation (SD) of placebo (mean slope riluzole – mean slope placebo/SD slopes placebo). To assess change within MSA and PSP strata and overall, we used the standardized response mean (SRM) defined as the ratio of the mean score change to the standard deviation (SD) of the score change. The SRM and ES values were interpreted as small (0.20 to 0.49), moderate (0.50 to 0.79) or large (>0.80) [38].

For power calculations and assessment of scale efficiency, sample size estimates were calculated within MSA and PSP strata and overall (p (α) = 0.05, p(1−β) = 0.80), using the total NNIPPS-PPS score slopes expressed as annual rate of change, those of the UPDRS motor score and SEADL, and those reported for UMSARS [39] and PSP-RS [5].

To explore the dimensional profiles of PSP and MSA, means and SD of scale scores at entry or of score slopes of change were calculated for the overall population and broken down by diagnostic strata (PSP versus MSA); Within diagnostic strata, these were tested for significance with Student's t test comparing means to a theoretical value of 0, and across diagnostic strata, with Student's t test for independent groups. For graphical representation of severity profiles at entry and at follow-up, mean dimensional scores at entry and mean slopes of change were expressed as percent of maximum dimensional scores. To assess the relative contribution of each dimension to overall severity within each disease, mean dimensional scores (at entry) and dimensional slopes of change were also expressed as percent of total score and of total score slope of change, respectively.

All analyses were conducted on the Intent to Treat population (ITT, or sub-groups of the ITT where appropriate), using SAS (9.1.1) software. Significance level was set at p<0.05 (2-sided), except when comparing dimensional sub scores between groups, where Bonferroni correction for multiple comparisons was applied (p<0.003).

## Results

The characteristics of the trial population and main results are reported in detail elsewhere [8]. The NNIPPS diagnostic criteria, validated prospectively against pathology, proved highly sensitive and specific, and the NNIPPS sample was broadly representative of the PSP and MSA patient population. Patients alive at the end of the study had at least 30 months follow-up and a total of 342 patients deceased during the trial (47% PSP patients, 43% MSA patients). Disease severity was comparable in both treatment groups at entry. On follow-up, since there was no treatment effect, on any primary or secondary efficacy measures, data from placebo and riluzole groups were combined.

### Face and content validity

All items of the scale were clearly understood by trial investigators, and considered appropriate to measure severity of PSP and MSA syndromes. The expert neurologists advised that all relevant dimensions for assessment of severity of both diseases were reasonably well represented with the items selected.

### Construct validity

**Figure 1:**
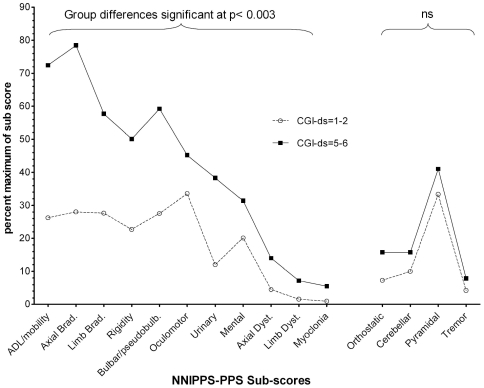
Dimensional scores of the PPS according to disease severity. Dimensional sub scores are expressed as percentage of the maximum possible score in the dimension (as indicated in Table 1, far left column). Comparisons (Student's t tests) were made between the two sub-groups defined by the extreme values of the Clinician Global Impression of disease severity (CGI-ds) in the overall study population. CGI Borderline/Mild illness (score 1–2) n = 93, dotted line; CGI Severe/extremely severe illness (score 5–6) n = 142, solid line. ns: not significant at p<0.003 with Bonferroni correction.

Due to poor rate of completion, the item “erectile dysfunction” was excluded from the scale prior to analysis. For the Principal Component Analysis (PCA), patients with any additional item missing (11% of cases) were excluded. The analysis population included complete records of 675 patients (PSP n = 317; MSA n = 358). The Principal Component Analysis (PCA) extracted 15 factors, altogether contributing to 62% of total variance (Table S1), with clearly identifiable clinical meaning, and corresponding to the *a priori* defined clinical dimensions. A single item, “sensory complaints” not correlating with any factor was further excluded from the scale. The first factor, consisting of two sets of items, 7 interview-based assessing activity of daily living and 7 from motor examination, was split for further analyses into two clinical dimensions *ADL/Mobility* and *Axial Bradykinesia* respectively. Items assessing tremor, correlating with 2 separate factors (*Tremor at rest* and *Postural tremor*), were combined into one single dimension (*Tremor*) as rest tremor symptoms were either absent or mild in these patients. The resulting 83-item scale, summarized into 15 dimensional sub scores and a total score, underwent thorough validation and was used to evaluate disease severity and progression. The internal consistency of the total score was excellent (Cronbach α = 0.92), and acceptable to high for all dimensional sub scores (Cronbach α = 0.68–0.94) except the *Pyramidal* score (Cronbach α = 0.39) (Table 1). Convergent validity was good as shown by the high correlation of the total score with global severity scales such as the CGI-ds (ρ = 0.72), HYS (ρ = 0.76) and SEADL (ρ = −0.80). Moderate correlation was found with Quality of Life scales (with PDQ-8: ρ = 0.48, SF-36 physical score ρ = −0.58). The *ADL/mobility*, *Axial* and *Limb bradykinesia*, and *Bulbar-pseudobulbar* sub scores were the most correlated (ρ = 0.49–0.85) with HYS, SEADL, and the CGI-ds (Table S2). Correlations of *Cerebellar*, *Pyramidal*, *Rigidity*, *Bulbar/pseudo bulbar*, *Mental*, *Limb Bradykinesia* and *Axial Bradykinesia*, *Orthostatic* and *Urinary* sub-scores with their corresponding VAS were satisfactory (ρ = 0.52–0.76). Correlations of the O*rthostatic* and *Urinary* scores with the CGI dysautonomia were also satisfactory (respectively ρ = 0.53 and 0.64). The *Mental* score correlated moderately with the FAB (ρ = −0.49) and the MMSE (ρ = −0.46). No relationship (ρ<0.30) with age or disease duration was found for any of the NNIPPS-PPS scores. This weak correlation with the disease duration could partly be explained by the bivariate distribution, with a substantial proportion of patients with low CGI-ds (1–3) in those with longer disease duration above 5 years (34%, i.e., slow progressors) and high CGI-ds (4–6) in those with short disease duration (<3 years) (37%, i.e., fast progressors). The convergent validity was further supported by the good discrimination between the two extreme groups of GCI-ds scores, with total score and 11 out 15 dimensional scores significantly higher (p<0.003 with Bonferroni correction) in the high severity group (Figure 1).

**Table 1:** NNIPPS-PPS scores at entry by diagnosis - Internal consistency and inter-rater reliability.

	PSP	MSA	Internal consistency	Inter-Rater Reliability
	N = 362	N = 398	N = 675	N = 116
Dimensional scores (range)	Mean ± SD	Mean ± SD	Cronbach's α	ICC
**ADL/Mobility (0–32)**	16.4±6.5^b^	15.0±6.6	0.87	0.93
**Axial bradykinesia (0–24)**	12.6±5.5	12.1±5.8	0.90	0.93
**Limb bradykinesia (0–32)**	12.8±6.8	13.7±6.8	0.93	0.85
**Rigidity (0–20)**	7.0±4.3	7.3±4.7	0.87	0.86
**Oculomotor (0–21)**	12.8±4.4^d^	2.3±2.7	0.94	0.87
**Cerebellar (0–24)**	1.0±1.8	5.1±5.1^d^	0.89	0.83
**Bulbar/Pseudo-bulbar (0–24)**	10.6±4.4^d^	9.2±4.4	0.82	0.89
**Mental (0–38)**	11.6±5.9^d^	7.5±5.0	0.78	0.77
**Orthostatic (0–12)**	0.7±2.0	2.7±3.5^d^	0.85	0.82
**Urinary (0–10)**	1.6±2.0	3.7±2.9^d^	0.68	0.93
**Limb dystonia (0–16)**	0.6±1.4	0.6±1.8	0.76	0.75
**Axial dystonia (0–12)**	1.1±2.1^a^	0.7±1.7	0.69	0.73
**Pyramidal (0–4)**	1.4±1.3	1.4±1.3	0.39	0.67
**Myoclonus (0–12)**	0.2±0.9	0.5±1.2^c^	0.75	0.54
**Tremor (0–28)**	1.3±2.1	2.4±3.3^d^	0.81	0.81
**Total score (0–309)**	91.6±30.4^b^	84.3±30.9	0.92	0.94

Means ± standard deviation (SD) of the NNIPPS-PPS dimensional and total scores at entry according to diagnosis: Progressive Supranuclear Palsy (PSP), Multiple System Atrophy (MSA).

All within groups comparisons (mean versus 0) are statistically significant (p<0.001 by Student's t test). Comparisons between diagnostic groups (Student's t test):

a p<0.05,

b p<0.01,

c p<0.001,

d p<0.0001.

Columns: Dimension labels (dimension ranges of scores- from 0 = normal, to maximum severity). Internal consistency is expressed as Cronbach's α coefficient, with threshold for acceptability set at α = 0.70. Agreement coefficients (Intra-Class Coefficients-ICC) from the inter-rater reliability study ranged from 0.54 (moderate) to 0.94 (excellent).

ADL = Activities of Daily Living.

**Figure 2:**
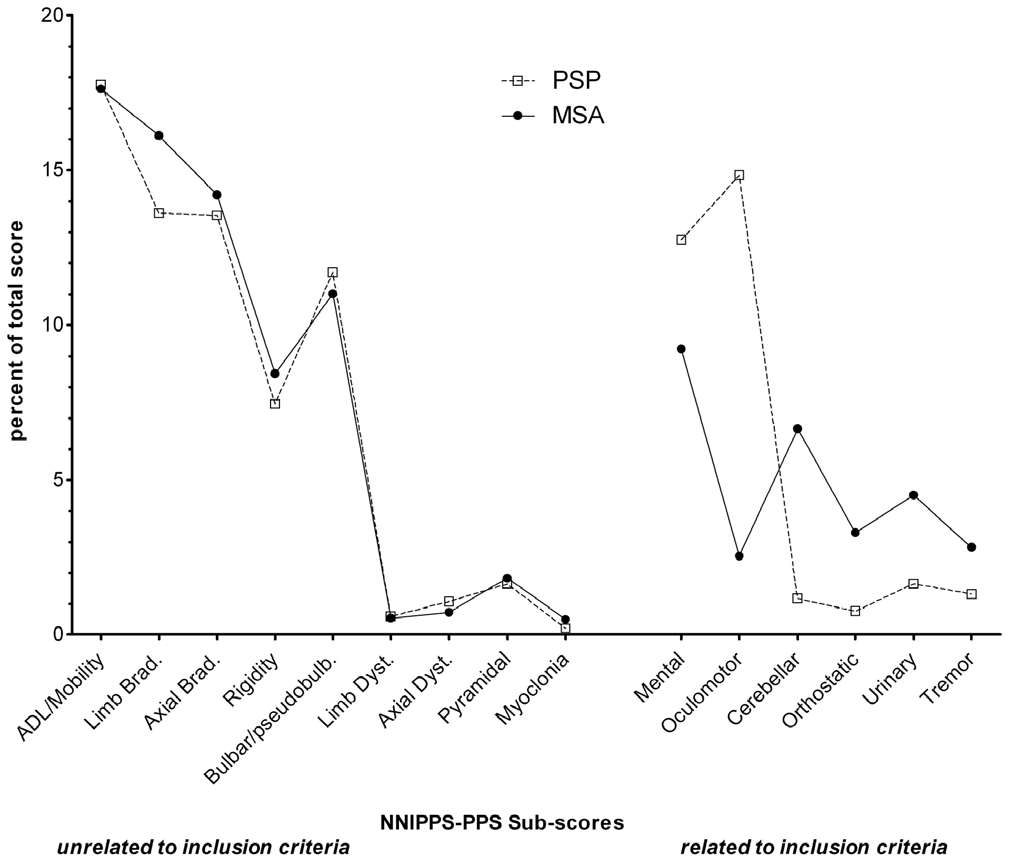
Dimensional profiles of PSP and MSA at entry. Overall profiles of Parkinson Plus Scale dimensional sub scores at entry for Progressive Supranuclear Palsy (PSP) and Multiple System Atrophy (MSA). Dimensional sub scores are expressed as percentage of the total score to evaluate relative contribution of each dimension to overall severity score. Comparisons (Student's t tests) were made between the two strata. PSP n = 362, dotted line; MSA n = 398, solid line. **Left:** sub scores unrelated to strata inclusion/exclusion criteria- three comparisons reached significance level at p<0.003: *Limb bradykinesia*, *Rigidity* and *Myoclonia* cumulating to 3.4% overall difference in contribution to total score. **Right:** sub scores related to strata inclusion/exclusion criteria- all differences are significant at p<0.003 with 28.2% overall difference in contribution to total score. Contributions of dimensions related to inclusion criteria amount for 27.6% and 17.3% for PSP and MSA respectively; Contributions of dimensions related to exclusion criteria amount for 4.9% and 11.8% in PSP and MSA respectively.

**Figure 3:**
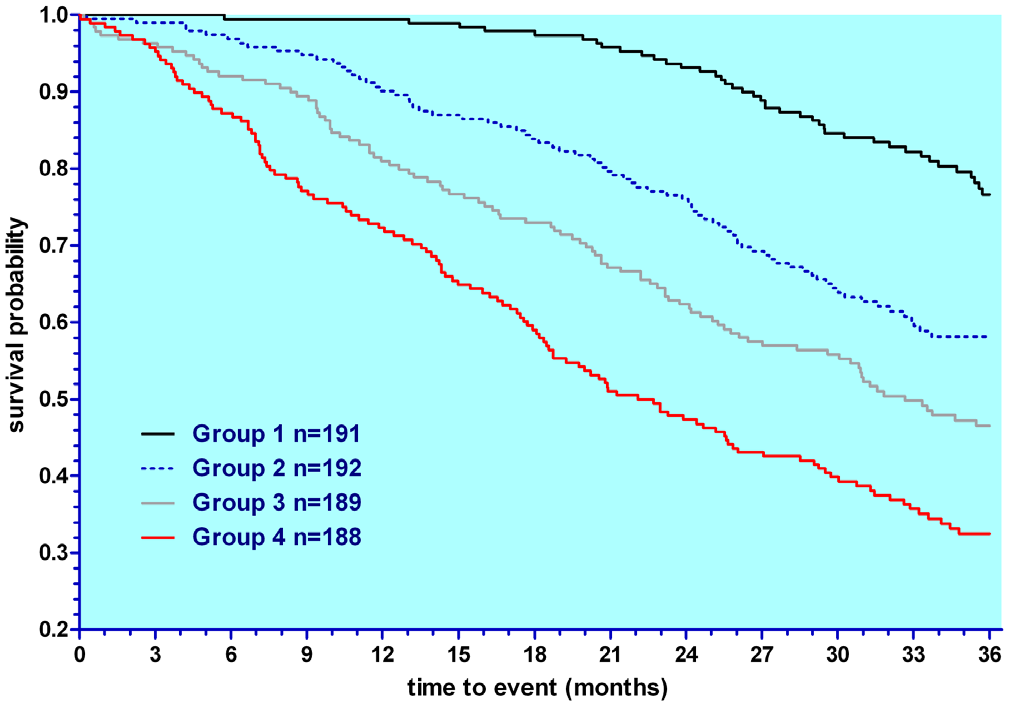
Predictive validity: 3-year survival according to NNIPPS-PPS total score at entry. Kaplan-Meier plot of the NNIPPS population broken down by quartiles of the NNIPPS-PPS total score at entry (grouping from lowest to highest severity: Group 1 score [0–65], Group 2 score [66–86], group 3 score [87–109], Group 4 score [110–182]. Log-rank analysis showed a highly significant difference (p<0.0001) between the four score groups with an inversely and linearly ordered survival according to score demonstrating an excellent predictive value of the NNIPPS-PPS.

The total score showed PSP patients to be slightly more severe at entry than MSA patients (Table 1). As inevitable in view of our strata inclusion and exclusion criteria, *Oculomotor* and *Mental* scores were higher in PSP, while MSA patients showed higher scores for *Tremor*, *Cerebellar*, *Orthostatic* and *Urinary* symptoms (Figure 2 right). When sub scores were expressed as percent of total score, for those scores unrelated to inclusion/exclusion criteria (n = 9) which contributed to approximately 70 percent of the total score, dimensional profiles were identical (Figure 2 Left). Importantly, within each diagnostic stratum, all mean dimensional sub-scores of the NNIPPS-PPS, including those related to strata inclusion and exclusion criteria were significantly different from zero (p<0.001 by Student's t test), indicating that all clinical dimensions were present in each disorder, although at varying levels (Table 1). Predictive validity of the total score at inclusion was confirmed by its strong relation to survival as shown by the univariate Cox model analysis (relative risk [95% CI] per point score = 1.07 [1.014–1.021] p<0.0001). On splitting the sample by quartiles, survival curves for the four groups were linearly separated (Figure 3), such that higher scores were associated with a worse prognosis. Among the 15 dimensional sub-scores, all except six (*Cerebellar*, *Mental*, *Limb dystonia*, *Myoclonus*, *Tremor*, *Pyramidal*) were significantly related to survival (Table 2). Multivariate stepwise Cox model analysis with candidate covariates including baseline demographic characteristics (strata, gender, disease duration, age at inclusion, age at onset), global severity scales (HYS, SEADL, CGI-ds, CGI dysautonomia) and NNIPPS-PPS total score, showed the latter as best predictor of survival (Table S3).

**Table 2:** Predictive validity of the NNIPPS-PPS total and dimensional scores on survival.

NNIPPS-PPS scores	RR [95%CI]	Khi^2^	P-Value
Total Score	1.017 [1.014–1.020]	105.42	<0.0001
Bulbar/Pseudo-bulbar	1.118 [1.093–1.144]	92.37	<0.0001
ADL/Mobility	1.084 [1.066–1.102]	91.96	<0.0001
Axial Bradykinesia	1.088 [1.068–1.109]	76.55	<0.0001
Urinary	1.149 [1.109–1.190]	59.72	<0.0001
Limb Bradykinesia	1.046 [1.030–1.062]	34.01	<0.0001
Rigidity	1.066 [1.042–1.091]	29.78	<0.0001
Axial Dystonia	1.121 [1.069–1.176]	22.21	<0.0001
Oculomotor	1.037 [1.020–1.055]	18.61	<0.0001
Orthostatic	1.069 [1.037–1.103]	17.82	<0.0001
Myoclonus	1.117 [1.027–1.215]	6.65	0.0099
Mental	1.023 [1.005–1.041]	6.13	0.0133
Pyramidal	1.073 [0.990–1.164]	2.94	0.0866
Cerebellar	1.019 [0.996–1.043]	2.65	0.1037
Limb Dystonia	1.041 [0.977–1.109]	1.55	0.2133
Tremor	1.006 [0.971–1.043]	0.12	0.7325

Univariate Cox model survival analysis. NNIPPS-PPS total and dimensional scores, Relative Risks per point score (RR) [95% confidence Interval] by descending Khi^2^. Intent to treat study population n = 760. Nine out of fifteen dimensions were significantly predictive of survival (at p<0.003 with Bonferroni correction).

### Inter-rater reliability

A total of 116 patients (MSA n = 74, PSP n = 42) were analyzed with a total of 33 evaluators including general neurologists, geriatricians, as well as experts in movement disorders. The characteristics of the 116 patients studied (France (n = 70), UK (n = 18) and Germany (n = 28)) were representative of the overall NNIPPS ITT population [8] (Table 3). The reliability of the total score was excellent (ICC = 0.94). For 14 of the 15 dimensional sub-scores, ICC values were substantial to almost perfect and moderate for one (*Myoclonus*) (Table 1). Item wise, inter-rater agreement was considered as acceptable (κ_w_>0.40, moderate to almost perfect) for 79 items (95%), including substantial for 38 items (κ_w_>0.6) and moderate for 41 (κ_w_ 0.4 to 0.6); four items had slight to fair reliability (κ_w_<0.4), two in the tremor section and two myoclonus items. On feedback, discrepancies between investigators' scores were accounted for (i) fluctuations in the severity of clinical symptoms and signs during the day, (ii) differences in interview technique, (iii) scoring of signs such as dystonia or myoclonus requiring expertise to be detected, and (iv) interpretation of items (mainly those of the mental function). Based on this feedback, standard operating procedures were established and implemented in the clinical trial.

**Table 3:** Patients Characteristics - Inter-rater reliability study.

	PSP	MSA	TOTAL
	N = 42	N = 74	N = 116
GENDER (F)	46%	45%	46%
AGE (years)	69±6	64±8	66±8
AGE AT ONSET (years) (36–77)	65±7	59±9	61±8
DISEASE DURATION (years )	3.9±1.8	5.0±1.9	4.6±2.0
1–2	31%	13%	19%
3–5	52%	49%	50%
6–8	14%	35%	27%
>8	2%	4%	4%
CGI disease severity (1–6)	3.5±1.0	3.7±.09	3.6±1.0
Mild/Moderately ill	48%	45%	46%
Markedly ill	31%	36%	34%
Severely/Extremely ill	21%	19%	20%
MODIFIED HOEHN & YAHR (0–5) Stage 2/2.5	24%	13%	17%
Stage 3	33%	29%	31%
Stage 4	12%	35%	26%
Stage 5	31%	24%	26%
SCHWAB & ENGLAND Activities of Daily living (0–100%)	54±27	49±24	50±25

Clinical characteristics of the 116 patients who took part to the inter-rater reliability study. Patients were recruited from 11 centers within the 3 participating countries to the NNIPPS study. Characteristics of these patients were close to those of the overall population (n = 760) [8].

CGI = Clinical Global Impression; PSP = Progressive Supranuclear Palsy; MSA = Multiple System Atrophy.

### Responsiveness

**Figure 4:**
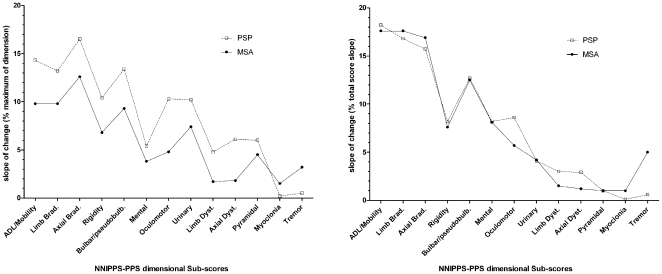
Profiles of PSP and MSA rates of change in dimensional sub scores. **Left figure:** Slopes of change in dimensional sub scores (excluding *Cerebellar* and *Orthostatic* sub scores) were expressed as percentage of the maximum possible score in the dimension. Progressive Supranuclear Palsy (PSP), n = 362, dotted line; Multiple System Atrophy (MSA), n = 398, solid line. PSP patients showed higher rates of progression in all but two sub scores (*Myoclonia* and *Tremor*) compared to MSA patients. **Right figure:** For each strata, slopes of change in dimensional sub scores were expressed as percentage of the total score slope of change (excluding *Cerebellar* and *Orthostatic* sub scores) to evaluate relative contribution of each dimension to overall severity progression rate. PSP n = 362, dotted line; MSA n = 398, solid line. PSP and MSA showed similar profiles for severity progression with a 15.3% cumulative difference in contribution of dimensions to overall slope, including dimensions related to inclusion/exclusion criteria (*Oculomotor*, *Mental*, *Urinary, and Tremor*). In both diseases, the *Akineto-Rigid* and *Bulbar* syndromes were those contributing most to overall severity progression (71.6% and 72.2% for PSP and MSA respectively).

There were 642 patients with at least two usable assessments (PSP n = 305, MSA n = 337) to assess rates of change. In both groups, the rate of change of the total score was highly significant (p<10^−4^), with PSP patients showing a higher progression rate as compared to MSA (p<10^−4^). In the PSP group, rates of change were highly significant (p<10^−4^) for all but three dimensions (*Orthostatic*, *Myoclonia*, *Tremor*) and in the MSA group one only (*Orthostatic*) was not significant (Table 4). In both groups the rate of change in *Orthostatic* score paradoxically showed non-significant improvement with time, which upon examination was found related to biased scorings for patients not being able to stand or walk anymore. The same bias was found to significantly affect *Cerebellar* scores at follow-up. The total score re-calculated without these two sub-scores revealed little alteration of the slope of change (Table 4). While there were clear differences in rates of progression for dimensional sub-scores between PSP and MSA (Figure 4 Left), when dimensional slopes of change within disease were expressed as percent of the total score slope, the profile of contribution of these to overall disease severity progression was remarkably similar even for dimensions related to inclusion criteria such as *Mental* or *Urinary* dimensions (Figure 4 Right).

**Table 4:** Responsiveness - slopes of change (mean ± SD) of the NNIPPS-PPS scores by Strata.

	PSP	Slope test	MSA	Slope test	Strata
Dimensional scores (max)	N = 305	P-value	N = 337	P-value	P-value
**ADL/Mobility (32)**	4.6±4.4	0.00001	3.1±4.0	0.00001	0.0001
**Axial bradykinesia (24)**	4.0±4.2	0.00001	3.0±3.4	0.00001	0.002
**Limb bradykinesia(32)**	4.2±5.9	0.00001	3.1±4.8	0.00001	0.007
**Rigidity (20)**	2.1±4.3	0.00001	1.4±3.2	0.00001	0.03
**Oculomotor (21)**	2.2±2.7	0.00001	1.0±2.8	0.00001	0.00001
**Cerebellar (24)**	0.8±3.1	0.0001	0.9±3.0	0.00001	0.92
**Bulbar/Pseudo-bulbar (24)**	3.2±3.1	0.00001	2.2±2.6	0.00001	0.00001
**Mental (38)**	2.1±4.5	0.00001	1.4±3.4	0.00001	0.05
**Orthostatic (12)**	−0.1±2.0	0.25	−0.22±2.8	0.15	0.66
**Urinary (10)**	1.0±2.1	0.00001	0.8±2.1	0.00001	0.08
**Limb dystonia (16)**	0.8±2.0	0.00001	0.3±1.7	0.005	0.0006
**Axial dystonia (12)**	0.7±1.9	0.00001	0.2±1.2	0.0008	0.0001
**Pyramidal (4)**	0.2±1.1	0.00001	0.2±1.0	0.001	0.61
**Myoclonia (12)**	0.02±1.1	0.52	0.2±1.5	0.02	0.14
**Tremor (28)**	0.1±2.7	0.94	0.9±3.5	0.00001	0.001
**Total score (309)**	25.8±20.8	0.00001	18.5±18.8	0.00001	0.00001
**Total score – 2** * **(273)**	25.2±20.1	0.00001	17.8±17.8	0.00001	0.00001

**PSP and MSA columns:** NNIPPS-PPS dimensional and total scores slopes of change (mean ± SD points per year) by strata; N: number of patients with at least two usable assessments over the three year follow-up. Maximum (most severe) theoretical scores are indicated in the far left column (brackets).

**Strata p value column:** p value from ANOVA comparing slope of change between strata.

**Slope test columns:** p value from within-group t test comparing slopes of change within strata (PSP, MSA) to 0 (no change).

*Total score-2: *Cerebellar* and *Orthostatic* scores at follow-up visits were found to be highly biased by interference with walking ability (some items becoming impossible to rate when the patient was unable to stand), and/or motor disability (eg, rigidity), their respective scorings were removed from this Total score calculation with minor alteration in the overall PPS slope of change in both groups.

There was no difference in the slope of change of the total score across the different levels of the CGI-ds (21.8 point per year in the lowest severity group versus 22.1 in the highest severity group, p = ns) indicating consistency of the scale across disease stages. Moreover there was no correlation between the baseline total score and slope of change (Spearman ρ = 0.04, p = ns).

Consistent with the lack of overall treatment effect on survival or on other functional scales [8], no difference was found between treatment groups for mean slopes of change in total NNIPPS-PPS score (Effect Size = 0.03).

When calculated across all visits, the standardized response mean (SRM) was large for both conditions (1.10 overall) with a higher response for PSP patients (SRM = 1.25) than for MSA patients (SRM = 1.00) thus confirming the more rapid progression in the former.

Compared to UPDRS, SEADL, UMSARS or PSP-RS, sample size estimates to detect a significant treatment difference in slope were substantially lower (30% to 60%) with the NNIPPS-PPS total score, whatever the group of patients considered (Table 5).

**Table 5:** Sample size estimates (per group) using change in slope of clinical scales.

Patients	Parameters	NNIPPS- PPS	SEADL	UPDRS 3	PSP-RS	UMSARS
All	*Mean slope ± SD*	*21.3±19.3*	*13.9±15.3*	*9.4±10.8*	**-**	**-**
	Change in slope 30%	144	212	229	**-**	**-**
	40%	80	118	129	**-**	**-**
	50%	51	76	82	-	-
PSP	*Mean slope ± SD*	*25.2±20.1*	*15.7±15.9*	*10.5±11.5*	*8.7±10.9*	-
	Change in slope 30%	112	179	209	274	-
	40%	63	102	118	155	-
	50%	40	65	76	100	-
MSA	*Mean slope ± SD*	*17.8±17.8*	*12.1±14.6*	*8.4±10.0*	*-*	*7.1±8.5*
	Change in slope 30%	174	253	250	*-*	251
	40%	97	142	140	*-*	141
	50%	62	92	90	*-*	91

Figures in cells are number of patients to be included per group, at α (2-tailed) = 0.05 and 1−β = 0.80, for different levels (30, 40, 50%) of expected change difference between groups on annual rate of change. Estimates for PSP, MSA or both groups combined, are based on effect sizes of NNIPPS-PPS, SEADL and UPDRS motor score (UPDRS-3) from the NNIPPS study, UMSARS [39] and PSP-RS [5]. Mean slopes ± SD as annual rate of change (points per year) are reported for each scale within each population.

**Abbreviations:** PPS = Parkinson Plus scale; SEADL = Schwab & England Activities of Daily Living scale; UPDRS-3 = Unified Parkinson Disease Rating Scale (Motor examination score); PSP-RS = Progressive Supranuclear Palsy Rating Scale; UMSARS = Unified Multiple System Atrophy Rating Scale.

## Discussion

The NNIPPS-PPS project is unique in attempting to develop and validate prospectively a comprehensive rating scale for both PSP and MSA that can be applied in the early stages of disease when sensitivity and specificity of current consensus diagnostic criteria are poor [2] or as yet untested [2], [40]. The validation of the NNIPPS-PPS scale in a large multicentre clinical trial in PSP and MSA enabled us to prospectively describe and compare symptoms severity and progression of a population of well characterised patients in which diagnostic criteria, prospectively tested against pathology, were both highly sensitive and specific [8]. Although the research criteria for inclusion in NNIPPS may differ from criteria for diagnosis in the clinic (e.g., patients with a pure cerebellar or pure autonomic presentation of MSA, and patients with PSP developing supranuclear palsy later in disease evolution, were formally excluded from the trial), our inclusion criteria were quite liberal. For example, we accepted a very mild akinetic-rigidity syndrome (i.e., only one of 14 items rated as mild in the UPDRS motor examination) [8]. On the whole, our sample should be relatively close to the clinical population, presenting a broad spectrum of severity and clinical profiles, thus allowing robust generalisation of the results.

The 15 dimensional sub-scores identified through factor analysis confirmed the hypothesised clinical dimensions, accurately reflecting the complex clinical profile of these two conditions. Overall, the dimensional scores at entry demonstrated a remarkably similar clinical profile in PSP and MSA, with complete overlap in nine dimensions (Figure 2 Left), together contributing to about 70% of the total severity score at entry in each disease. These findings are well supported by the psychometric quality of the scale to measure disease severity, in terms of reliability, construct validity, predictivity and sensitivity to change.

Although the data were acquired in the setting of a’ field-type’ study involving numerous clinicians, inter-rater reliability of the NNIPPS-PPS was high, both at the item level and sub-scores with 95% and 87% with acceptable to high agreement, respectively. Likewise, total score and all dimensions except the *Pyramidal* one showed acceptable to high internal consistency.

For assessment of convergent validity, we chose several generic evaluations to investigate different approaches of severity assessment. The scale demonstrated a good convergence with other clinical measures for the overall score and for dimensions where reference measures could be obtained. Predictive validity of the scale was clearly demonstrated through survival analysis with total score and most dimensional scores highly predictive of survival.

Analysis of the repeated measures over the 3 year follow-up showed that the scale appropriately reflects disease progression (Table 4), except for *Myoclonia* which had a very low frequency and low severity in both conditions, and the *Cerebellar* and *Orthostatic* dimensions which could not be reliably assessed at follow-up once patients were unable to stand, or were treated for orthostatic symptoms. On the whole, the slopes of progression of sub scores also demonstrated a remarkably similar profile in MSA and PSP (Figure 4 Right). Nevertheless, as previously reported in [8], PSP patients had more severe symptoms and signs at entry, and had a faster rate of progression on follow-up compared to MSA in terms of both functional disability and survival. This difference was clearly detected with the NNIPPS-PPS scale, demonstrating the good psychometric quality of the scale (Figure 4 Left). To confirm the usefulness of the total score as an outcome measure for clinical interventions, we calculated the standardized response mean (SRM) which reflects the ability of the scale to detect change. The NNIPPS-PPS total score was able to detect a smaller effect for disease progression than we originally hypothesized [8]. Compared to UPDRS, SEADL, PSP-RS or UMSAR scales, the NNIPPS-PPS scale requires fewer patients to detect a given treatment effect. However, the absence of a treatment effect with riluzole precluded the assessment of responsiveness to treatment [8].

A major concern for the application of any scale is the relation between rate of progression and disease severity (i.e., linearity). Non-linearity contributes to bias as the slope varies with disease severity. We found no correlation between total score slope and the total score at inclusion, or between slope and CGI-ds, as the annual decrease remained constant across the different severity levels, from mild to very severe. This is at variance with the SEADL for which the annual rate of progression decreased with greater disease severity (data not shown), or with the UMSARS [39]. This may be explained by a ceiling effect affecting these measurements, which was not present with the NNIPPS-PPS.

Several dimensions, *Dystonia* (*axial* or *Limb*), *Myoclonia*, *Cerebellar*, *Orthostatic* and *Pyramidal* provided limited information. Although not frequent and not contributing much to overall disease severity in our analysis, *Dystonia* and *Myoclonia* dimensions showed acceptable psychometric properties and should be kept as they may be disabling, of prognostic value when present and diagnostically useful. *Cerebellar* and *Orthostatic* dimensions showed acceptable construct validity and reliability but their assessments were biased at follow-up, suggesting the need for revised standard operating procedures. The *Pyramidal* dimension proved difficult to quantify, had low internal consistency and reliability, hence its contribution to overall disease severity and progression is questionable. However, nearly 50% of patients in both conditions presented with pyramidal signs at inclusion [8]. To assess its real contribution to disease severity and progression the construct of the *Pyramidal* dimension should be reconsidered. Lastly, the domain exploring sexual symptoms requires further development to complete evaluation of dysautonomia. As it is likely that PSP could be combined with other tauopathies such as corticobasal degeneration syndrome (CBD), further work on the scale may consider adapting the scale for CBD, including elements such as apraxia. These issues are now being addressed in a new ongoing study.

The development of a scale should allow an ‘unbiased’ assessment of the full range of functional deficits in the disorders in question. This is particularly important in complex multisystem disorders such as PSP and MSA. We chose to design a comprehensive, more extended scale, rather than to limit the dimensions to the most characteristic features of PSP and MSA. In that respect, we have confirmed that the *Bulbar* syndrome is an independent dimension with important contributions to disease severity, progression and prognosis in both conditions. While cerebellar dysfunction is characteristic of MSA, it also occurs in PSP, as Steele et al. [41] pointed in their original description of PSP. Likewise, cognitive abnormalities have often been regarded as unimportant in MSA [18], but we have shown that these are relatively common in MSA. In a previous paper [42], we showed that cognitive impairment substantially increased the false diagnosis rate in the MSA group. However, the overall rate of false diagnosis was low (12%) [8] and the cognitive impairment predicted only a third of these. Thus, these few misdiagnosed cases cannot account for the decline in mental functioning in patients diagnosed with MSA. Furthermore, 18.2% of the neuropathologically confirmed MSA cases were found to be cognitively impaired a frequency similar to the trial population (i.e., 20%). Although generally less severe than in PSP, the profile of cognitive dysfunction in MSA was similar on the Dementia Rating Scale [42]. Our results confirm that all *a priori* defined dimensions are present in both disorders, differing only in terms of degree of severity or rate of progression.

Overall, the NNIPPS study has provided new insights on the natural history of PSP and MSA. Our assumptions at the planning stage were that overall diagnostic accuracy would be low, particularly early in the disease course and that some overlap might therefore be present in the assessments of disease severity. Our results have shown that the NNIPPS diagnostic criteria had good sensitivity and specificity even at the early stage [8], while the dimensional profile of disease severity and progression as analyzed here showed wider overlap than expected. These findings are not contradictory as the NNIPPS diagnostic criteria though specific to each condition represented only a partial aspect of the overall disease severity assessed with the NNIPS-PPS. On the other hand, our consistent findings of similar patterns of cognitive disability in MSA and PSP [42] and their high contribution to overall disease severity and progression, argue strongly that the current consensus criteria for MSA [40] should be revised [43], [44].

In **conclusion**, we have developed a clinical scale combining features of MSA and PSP, which in the early stages share common features, making accurate diagnosis difficult. The study has provided evidence, prospectively collected in a large multicentre cohort that there is consistent overlap between these disorders, differing in degree of severity and progression rates. Our results show that the NNIPPS-PPS has the psychometric qualities required to measure disease severity and progression in both diseases, is efficient for powering trials, and is strongly predictive of survival. These features make it suitable for capturing the effect of disease-modifying therapy in clinical trials for MSA, PSP or aty pical parkinsonian (‘parkinson plus’) syndromes generically.

## Supporting Information

Supporting information S1 **PARKINSON PLUS SCALE (NNIPPS-PPS) (83 items).** The 83 items of the NNIPPS-PPS scale are presented within their respective dimensions along with scoring definition for each item.
(DOC)

Supporting information S2 **Details of psychometric validation methods and results.**
(DOC)

Table S1 **Factor analysis of the NNIPPS-Parkinson Plus Scale.** Data from 675 patients (317 PSP and 358 MSA) with fully completed scales at inclusion were submitted to Principal Component Analysis (PCA). The analysis included 84 out of the 85 items of the scale as one item, “erectile dysfunction”, could not be included due to poor completion rate. 85 patients (11%) had 1 to 10 item scores missing and were therefore excluded from analysis. Fifteen factors were extracted following varimax rotation. Loadings>0.30 of each item with corresponding factor are listed. Only one item, “*sensory complaints*”, did not correlate with any factor. For further analysis, and on clinical grounds, the first factor was split into 2 clinical dimensions: *ADL/mobility* based on interview items, and *Axial bradykinesia* based on motor examination; the 2 tremor factors were combined into a single dimension (*Tremor*).
(DOC)

Table S2 **Convergent validity.** Spearman rank correlations of the 15 dimensional and total scores of the NNIPPS Parkinson Plus scale at entry with other measures of clinical severity: the Clinical Global Impression Disease severity (CGI-ds), Hoehn & Yahr staging (HYS), Schwab & England scale (SEADL), Visual analog Scale (VAS) of severity of clinical syndromes including akineto-rigidity (AKIN.), dysautonomia (DYSAUT.), cerebellar (CEREB.), pyramidal (PYRAM.), bulbar/pseudobulbar (BULB.), cognitive (COG.) and behavioral (BEHAV.), and two measures of cognition, the Frontal Assessment Battery (FAB) and Mini Mental State Examination (MMSE). Moderate to high coefficients (≥0.40) are in bold characters. * Of note, *Oculomotor*, *Axial dystonia*, *Limb dystonia*, *Myoclonus* and *Tremor* had no reference measures. ADL = Activities of Daily Living
(DOC)

Table S3 **Predictive Validity - Multivariate stepwise Cox model survival analysis.** * Strata codes: 0 = PSP, 1 = MSA. Df = degrees of freedom, SD = standard deviation RR = relative risk, CI = confidence interval. Candidate covariates included *strata*, *gender*, *disease duration*, *age at inclusion*, *age at onset of symptoms*, *Hoehn & Yahr Staging*, *Schwab & England Activities of Daily Living*, *Clinician Global Impression (CGI)-disease severity and Clinician Global Impression (CGI)-Dysautonomia score*. The stepwise Cox model retained (by order of entry), the *NNIPPS-PPS total score*, *CGI-dysautonomia*, *Disease duration*, *CGI-disease severity*, and *Strata* as the best set of independent predictors.
(DOC)

## References

[1] Litvan I, Mangone CA, McKee A, Verny M, Parsa A (1996). Natural history of progressive supranuclear palsy (Steele-Richardson-Olszewski syndrome) and clinical predictors of survival: a clinicopathological study.. J Neurol Neurosurg Psychiatry.

[2] Litvan I, Bhatia KP, Burn DJ, Goetz CG, Lang AE (2003). Movement Disorders Society Scientific Issues Committee report: SIC Task Force appraisal of clinical diagnostic criteria for Parkinsonian disorders.. Mov Disord.

[3] Testa D, Monza D, Ferrarini M, Soliveri P, Girotti F (2001). Comparison of natural histories of progressive supranuclear palsy and multiple system atrophy.. Neurol Sci.

[4] Ben-Shlomo Y, Wenning GK, Tison F, Quinn NP (1997). Survival of patients with pathologically proven multiple system atrophy: a meta-analysis.. Neurology.

[5] Golbe LI, Ohman-Strickland PA (2007). A clinical rating scale for progressive supranuclear palsy.. Brain.

[6] Schrag A, Ben-Shlomo Y, Quinn NP (1999). Prevalence of progressive supranuclear palsy and multiple system atrophy: a cross-sectional study.. Lancet.

[7] Schrag A, Wenning GK, Quinn N, Ben-Shlomo Y (2008). Survival in multiple system atrophy.. Mov Disord.

[8] Bensimon G, Ludolph A, Agid Y, Vidailhet M, Payan C (2009). Riluzole treatment, survival and diagnostic criteria in Parkinson plus disorders: The NNIPPS Study.. Brain.

[9] Robbins TW, James M, Owen AM, Lange KW, Lees AJ (1994). Cognitive deficits in progressive supranuclear palsy, Parkinson's disease and multiple system atrophy in tests sensitive to frontal lobe dysfunction.. J Neurol Neurosurg Psychiatry.

[10] Burk K, Daum I, Rub U (2006). Cognitive function in multiple system atrophy of the cerebellar type.. Mov Disord.

[11] Kroonenberg PM, Oort FJ, Stebbins GT, Leurgans SE, Cubo E (2006). Motor function in Parkinson's disease and supranuclear palsy: simultaneous factor analysis of a clinical scale in several populations.. BMC Med Res Methodol.

[12] Lyoo CH, Jeong Y, Ryu YH, Lee SY, Song TJ (2008). Effects of disease duration on the clinical features and brain glucose metabolism in patients with mixed type multiple system atrophy.. Brain.

[13] Fahn S, Elton RL, Fahn S, Marsden CD, Calne D, Goldstein M, UPDRS program members (1987). Unified Parkinson's disease rating scale.. Recent developments in Parkinson's disease. Vol 2.

[14] Cubo E, Stebbins GT, Golbe LI, Nieves AV, Leurgans S (2000). Application of the Unified Parkinson's Disease Rating Scale in Progressive Supranuclear Palsy: Factor Analysis of the motor scale.. Mov Disord.

[15] Seppi K, Yekhlef F, Diem A, Luginger Wolf E, Mueller J (2005). Progression of parkinsonism in multiple system atrophy.. J Neurol.

[16] Tison F, Yekhlef F, Chrysostome V, Balestre E, Quinn NP (2002). Parkinsonism in multiple system atrophy: natural history, severity (UPDRS-III), and disability assessment compared with Parkinson's disease.. Mov Disord.

[17] Golbe LI, Lepore FE, Johnson WG, Belsh JM, Powell AL (1999). Inter-rater reliability of the Progressive Supranuclear Palsy Rating Scale.. Neurology.

[18] Wenning GK, Tison F, Seppi K, Sampaio C, Diem A (2004). Multiple System Atrophy Study Group. Development and validation of the Unified Multiple System Atrophy Rating Scale (UMSARS).. Mov Disord.

[19] Geser F, Wenning GK, Seppi K, Stampfer-Kountchev M, Scherfler C (2006). Progression of multiple system atrophy (MSA): a prospective natural history study by the European MSA Study Group (EMSA SG).. Mov Disord.

[20] Geser F, Seppi K, Stampfer-Kountchev M, Köllensperger M, Diem A (2005). The European Multiple System Atrophy-Study Group (EMSA-SG).. J Neural Transm.

[21] Trouillas P, TakayanagI T, Hallett M, Currier RD, Subramony SH (1997). The Ataxia Neuropharmacology Committee of the World Federation of Neurology. International Cooperative Ataxia Rating Scale for pharmacological assessment of the cerebellar syndrome.. J Neurol Sci.

[22] Kurtzke JF (1983). Rating neurologic impairment in multiple sclerosis: an expanded disability status scale (EDSS).. Neurology.

[23] Suarez GA, Opfer-Gehrking TL, Offord KP, Atkinson EJ, O'brien PC (1999). The Autonomic Symptom Profile: a new instrument to assess autonomic symptoms.. Neurology.

[24] American Psychological Association, American Educational Research Association, National Council on Measurement in Education (1999). Standards for educational and psychological testing.

[25] Nunnally JC, Bernstein IH (1994). Psychometric theory (3^rd^ ed.).

[26] Hoehn MM, Yahr MD (1967). Parkinsonism: onset, progression and mortality.. Neurology.

[27] Schwab R, England AJ, Gillingham FJ, Donaldson IML (1969). Projection techniques for evaluating surgery in Parkinson's Disease.. Third symposium on Parkinson's Disease, Royal College of Surgeons.

[28] Folstein MF, Folstein SE, McHugh PR (1975). “Mini Mental State”. A practical method for grading the cognitive state of patients for the clinician.. J Psychiatr Res.

[29] Dubois B, Slachevsky A, Litvan I, Pillon B (2000). The FAB: a frontal assessment battery at bedside.. Neurology.

[30] Jenkinson C, Peto V, Fitzpatrick R, Greenhall R, Hyman N (1997). The PDQ-8: development and validation of a short-form Parkinson's disease questionnaire.. Psychol Health.

[31] Brazier JE, Harper R, Jones NMB, O'Cathain A, Westlake L (1992). Validating the SF-36 health survey questionnaire: new outcome measure for primary care.. BMJ.

[32] Cox DR (1972). Regression models and life tables.. J R Stat Soc.

[33] Fleiss JL, Bradley RA, Hunter JS, Kendal DG, Watson GS (1981). The measurement of inter-rater agreement.. Statistical methods for rates and proportions (2^nd^ ed.).

[34] Cohen J (1960). A coefficient of agreement for nominal scales. Educational and psychological measurements..

[35] Shrout PE, Fleiss JL (1979). Intraclass correlations: uses in assessing rater reliability.. Psychol Bull.

[36] Sim J, Wright CC (2005). The Kappa Statistic in Reliability Studies: Use, Interpretation, and Sample Size Requirements.. Phys Ther.

[37] Wu MC, Bailey K (1988). Analysing change in the presence of informative right censoring caused by death and withdrawal, and staggered entry.. Control Clin Trials.

[38] Cohen J (1988). Statistical power analysis for the behavioral sciences (2^nd^ ed.).

[39] May S, Gilman S, Sowell BB, Thomas RG, Stern MB (2007). Potential outcome measures and trial design issues for multiple system atrophy.. Mov Disord.

[40] Gilman S, Wenning GK, Low PA, Brooks DJ, Mathias CJ (2008). Second consensus statement on the diagnosis of multiple system atrophy.. Neurology.

[41] Steele JC, Richardson JC, Olszewski J (1964). Progressive supranuclear palsy. A heterogeneous degeneration involving the brain stem, basal ganglia and cerebellum with vertical gaze and pseudobulbar palsy, nuchal dystonia and dementia.. Arch Neurol.

[42] Brown RG, Lacomblez L, Landwehrmeyer BG, Bak T, Uttner I (2010). Cognitive impairment in patients with multiple system atrophy and progressive supranuclear palsy.. Brain.

[43] Aerts M, Bloem B (2011). “This large observational study has generated unique and important new insights into the presence, severity…” Evaluation of: [Brown RG et al. Cognitive impairment in patients with multiple system atrophy and progressive supranuclear palsy. Brain. 2010 Aug; 133(Pt 8):2382–93; doi: 10.1093/brain/awq158].. http://F1000.com/4671975.

[44] Vanderhorst V, Tarsy D (2010). “This study demonstrates that cognitive problems occur in the majority of patients with progressive supranuclear…” Evaluation of: [Brown RG et al. Cognitive impairment in patients with multiple system atrophy and progressive supranuclear palsy. Brain. 2010 Aug; 133(Pt 8):2382–93; doi: 10.1093/brain/awq158].. http://F1000.com/4671975.

