# Supplementary material for: Disease Severity and Progression in Progressive Supranuclear Palsy and Multiple System Atrophy: Validation of the NNIPPS – PARKINSON PLUS SCALE
Source: PLoS One. 2011 Aug 4;6(8):e22293. doi: 10.1371/journal.pone.0022293 (PMC3150329; doi:10.1371/journal.pone.0022293)
Supplement: Supporting information S1 — PARKINSON PLUS SCALE (NNIPPS-PPS) (83 items). The 83 items of the NNIPPS-PPS scale are presented within their respective dimensions along with scoring definition for each item. (DOC) [file pone.0022293.s001.doc]

**Supporting information S1: PARKINSON PLUS SCALE (NNIPPS-PPS) (83 items)**

**NNIPPS-PPS** 1/6

| ***MENTAL FUNCTION (interview* *with patient or caregiver)*** | |
| --- | --- |
| ***1. Intellectual impairment***  0 None.  1 Mild. Consistent forgetfulness with partial recollection of events and no other difficulties.  2 Moderate memory loss, with disorientation and moderate difficulty in handling complex problems. Mild but definite impairment of function at home with need of occasional prompting.  3 Severe memory loss with disorientation for time and often to place. Severe impairment in handling problems.  4 Severe memory loss with orientation preserved to person only. Unable to make judgements or solve problems. Require much help with personal care. Cannot be left alone at all.  ***2. Thought disorder***  0 None.  1 Vivid dreaming.  2 « Benign » hallucination with insight retained.  3 Occasional to frequent hallucinations or delusions without insight; could interfere with daily activities.  4 Persistent hallucinations, delusions, or florid psychosis. Not able to care for self.  ***3. Depression***  0 Not present.  1 Periods of sadness or guilt greater than normal, never sustained for days or weeks.  2 Sustained depression (1 week or more).  3 Sustained depression with vegetative symptoms (insomnia, anorexia, weight loss, loss of interest).  4 Sustained depression with vegetative symptoms and suicidal thoughts or intent.  ***4. Motivation / Initiative***  0 Normal.  1 Less assertive than usual, more passive.  2 Loss of initiative or disinterest in elective (non-routine) activities.  3 Loss of initiative or disinterest in day-to-day (routine) activities.  4 Withdrawn, complete loss of motivation.  ***5. Aggressiveness***  0 No increase in aggressiveness.  1 Increased, but not interfering with family interactions.  2 Interfering with family interactions. | ***6. Loss of concentration / Integration ability***  0 Clearly absent.  1 Equivocal or minimal.  2 Clearly present, but not interfering with activities of daily living (ADL).  3 Interfering mildly with ADL.  4 Interfering markedly with ADL.  ***7. Bradyphrenia***  0 Clearly absent.  1 Equivocal or minimal.  2 Clearly present, but not interfering with activities of daily living (ADL).  3 Interfering mildly with ADL.  4 Interfering markedly with ADL.  ***8. Emotional incontinence***  0 Clearly absent.  1 Equivocal or minimal.  2 Clearly present, but not interfering with activities of daily living (ADL).  3 Interfering mildly with ADL.  4 Interfering markedly with ADL.  ***9. Withdrawal***  0 Clearly absent.  1 Equivocal or minimal.  2 Clearly present, but not interfering with activities of daily living (ADL).  3 Interfering mildly with ADL.  4 Interfering markedly with ADL.  ***10. Grasping / imitative / Utilising behaviour***  0 Clearly absent.  1 Equivocal or minimal.  2 Clearly present, but not interfering with activities of daily living (ADL).  3 Interfering mildly with ADL.  4 Interfering markedly with ADL. |

**NNIPPS-PPS 2/6**

| ***BULBAR-PSEUDOBULBAR SIGNS*** | |
| --- | --- |
| ***1****.* ***Salivation***  0 Normal.  1 Slight but definite excess of saliva in mouth; may have night-time drooling.  2 Moderately excessive saliva; may have minimal drooling.  3 Marked excess of saliva with some drooling.  4 Marked drooling, requires constant tissues or handkerchief.  ***2. Swallowing***  0 Normal.  1 Rare choking.  2 Occasional choking.  3 Requires soft food.  4 Requires NG tube or gastrostomy feeding.  ***3. Speech (motor examination)***  0 Normal.  1 Slight loss of expression, dictation and/or volume.  2 Monotone, slurred but understandable; moderately impaired.  3 Marked impairment, difficult to understand.  4 Unintelligible. | ***4. Facial expression***  0 Normal.  1 Minimal hypomimia, could be normal « Poker Face ».  2 Slight but definitely abnormal diminution of facial expression.  3 Moderate hypomimia; lips parted some of the time.  4 Marked or fixed facies with severe or complete loss of expression; lips parted ¼ inch or more.  ***5.Cough***  0 Normal.  1 Slightly impaired.  2 Markedly impaired.  3 Weak.  4 Impossible or artificial measures required (suctioning, tracheotomy).  **6. Speech (ADL)**  0 Normal.  1 Mildly affected. No difficulty being understood.  2 Moderately affected. Sometimes asked to repeat statements.  3 Severely affected, Frequently asked to repeat statements.  4 Unintelligible most of the time. |
| ***ADL / MOBILITY (interview with patient or caregiver)*** | |
| ***1. Handwriting***  0 Normal.  1 Slightly slow or small.  2 Moderately slow or small ; all words are legible.  3 Severely affected; not all words are legible.  4 The majority of words are not legible.  ***2. Cutting food and handling utensils***  0 Normal.  1 Somewhat slow and clumsy, but no help needed.  2 Can cut most foods, although clumsy and slow; some help needed.  3 Food must be cut by someone, but can still feed slowly.  4 Needs to be fed.  ***3. Dressing***  0 Normal.  1 Somewhat slow, but no help needed.  2 Occasional assistance with buttoning, getting arms in sleeves.  3 Considerable help required, but can do some things alone.  4 Helpless.  ***4. Hygiene***  0 Normal.  1 Somewhat slow, but no help needed.  2 Needs help to shower or bathe ; or very slow in hygiene care.  3 Requires assistance for washing, brushing teeth, combing hair, going to bathroom.  4 Foley catheter or other mechanical aids. | ***5. Turning in bed and adjusting bed clothes***  0 Normal.  1 Somewhat slow and clumsy, but no help needed  2 Can turn alone or adjust sheets, but with great difficulty.  3 Can initiate, but not turn or adjust sheets alone.  4 Helpless.  ***6. Falling unrelated to freezing***  0 None.  1 Rare falling.  2 Occasional falls, less than once per day.  3 Falls an average of once a day.  4 Falls more than once daily or wheelchair bound due to falls  9 Wheelchair bound due to other reason (accident …),  specify : __________________________  ***7. Freezing when walking***  0 None.  1 Rare freezing when walking; may have start-hesitation.  2 Occasional freezing when walking.  3 Frequent freezing. Occasional falls from freezing  4 Frequent falls from freezing or wheelchair bound due to freezing  9 If not assessable, specify why : ***(example: MSA Cerebellar type)***  ***8. Walking***  0 Normal.  1 Mild difficulty. May not swing arms or may tend to drag legs.  2 Moderate difficulty, but requires little or no assistance.  3 Severe disturbances or walking, requiring assistance.  4 Cannot walk at all, even with assistance. |

**NNIPPS-PPS 3/6**

| ***TREMOR*** | ***RIGIDITY*** |
| --- | --- |
| ***1. Tremor/ADL***  0 Absent.  1 Slight and infrequently present.  2 Moderate, bothersome to patient  3 Severe ; interferes with many activities  4 Marked ; interferes with most activities.  ***2. Face, lips, chin tremor***  0 Absent.  1 Slight and infrequently present.  2 Mild in amplitude and persistent ; or moderate in amplitude, but only intermittently present.  3 Moderate in amplitude and present most of the time.  4 Marked in amplitude and present most of the time.  ***3. Right hand tremor at rest***  0 Absent.  1 Slight and infrequently present  2 Mild in amplitude and persistent ; or moderate in amplitude, but only intermittently present.  3 Moderate in amplitude and present most of the time.  4 Marked in amplitude and present most of the time.  ***4. Left hand tremor at rest***  0 Absent.  1 Slight and infrequently present.  2 Mild in amplitude and persistent ; or moderate in amplitude, but only intermittently present.  3 Moderate in amplitude and present most of the time.  4 Marked in amplitude and present most of the time.  ***5. Right foot tremor at rest***  0 Absent.  1 Slight and infrequently present.  2 Mild in amplitude and persistent ; or moderate in amplitude, but only intermittently present.  3 Moderate in amplitude and present most of the time.  4 Marked in amplitude and present most of the time.  ***6. Left foot tremor at rest***  0 Absent.  1 Slight and infrequently present.  2 Mild in amplitude and persistent ; or moderate in amplitude, but only intermittently present.  3 Moderate in amplitude and present most of the time.  4 Marked in amplitude and present most of the time.  ***7. Action or postural tremor of right hand***  0 Absent.  1 Slight ; present with action.  2 Moderate in amplitude, present with action.  3 Moderate in amplitude with posture holding as well as action.  4 Marked in amplitude ; interferes with feeding.  ***8. Action or postural tremor*** ***of left hand***  0 Absent.  1 Slight ; present with action.  2 Moderate in amplitude, present with action.  3 Moderate in amplitude with posture holding as well as action.  4 Marked in amplitude; interferes with feeding. | ***1. Neck rigidity***  0 Absent.  1 Slight or detectable only when activated by mirror or other movement.  2 Mild to moderate.  3 Marked, but full range of motion easily achieved.  4 Severe, range of motion achieved with difficulty.  ***2. Right upper extremity rigidity***  0 Absent.  1 Slight or detectable only when activated by mirror or other movement.  2 Mild to moderate.  3 Marked, but full range of motion easily achieved.  4 Severe, range of motion achieved with difficulty.  ***3. Left upper extremity rigidity***  0 Absent.  1 Slight or detectable only when activated by mirror or other movement.  2 Mild to moderate.  3 Marked, but full range of motion easily achieved.  4 Severe, range of motion achieved with difficulty.  ***4. Right lower extremity rigidity***  0 Absent.  1 Slight or detectable only when activated by mirror or other movement.  2 Mild to moderate.  3 Marked, but full range of motion easily achieved.  4 Severe, range of motion achieved with difficulty.  ***5. Left lower extremity rigidity***  0 Absent.  1 Slight or detectable only when activated by mirror or other movement.  2 Mild to moderate.  3 Marked, but full range of motion easily achieved.  4 Severe, range of motion achieved with difficulty. |
|  | ***MYOCLONUS*** |
|  | ***1. At rest***  0 Absent.  1 Mild.  2 Obvious.  3 Disabling.  4 Severe.  ***2. In action***  0 Absent.  1 Mild.  2 Obvious.  3 Disabling.  4 Severe.  ***3.With stimulation***  0 Absent.  1 Mild.  2 Obvious.  3 Disabling.  4 Severe. |

**NNIPPS-PPS 4/6**

| ***LIMB BRADYKINESIA*** | |
| --- | --- |
| ***1. Right fingers taps***  0 Normal.  1 Mild slowing and/or reduction in amplitude.  2 Moderately impaired. Definite and early fatiguing. May have occasional arrests in movement.  3 Severely impaired. Frequent hesitation in initiating movements or arrests in ongoing movement.  4 Can barely perform the task.  ***2. Left fingers taps***  0 Normal.  1 Mild slowing and/or reduction in amplitude.  2 Moderately impaired. Definite and early fatiguing. May have occasional arrests in movement.  3 Severely impaired. Frequent hesitation in initiating movements or arrests in ongoing movement.  4 Can barely perform the task.  ***3. Right hand movements***  0 Normal.  1 Mild slowing and/or reduction in amplitude.  2 Moderately impaired. Definite and early fatiguing. May have occasional arrests in movement.  3 Severely impaired. Frequent hesitation in initiating movements or arrests in ongoing movement.  4 Can barely perform the task.  ***4. Left hand movements***  0 Normal.  1 Mild slowing and/or reduction in amplitude.  2 Moderately impaired. Definite and early fatiguing. May have occasional arrests in movement.  3 Severely impaired. Frequent hesitation in initiating movements or arrests in ongoing movement.  4 Can barely perform the task. | ***5. Rapid alternating movements of the right hand***  0 Normal.  1 Mild slowing and/or reduction in amplitude.  2 Moderately impaired. Definite and early fatiguing. May have occasional arrests in movement.  3 Severely impaired. Frequent hesitation in initiating movements or arrests in ongoing movement.  4 Can barely perform the task.  ***6. Rapid alternating movements of the left hand***  0 Normal.  1 Mild slowing and/or reduction in amplitude.  2 Moderately impaired. Definite and early fatiguing. May have occasional arrests in movement.  3 Severely impaired. Frequent hesitation in initiating movements or arrests in ongoing movement.  4 Can barely perform the task.  ***7. Right Leg agility***  0 Normal.  1 Mild slowing and/or reduction in amplitude.  2 Moderately impaired. Definite and early fatiguing. May have occasional arrests in movement.  3 Severely impaired. Frequent hesitation in initiating movements or arrests in ongoing movement.  4 Can barely perform the task.  ***8. Left Leg agility***  0 Normal.  1 Mild slowing and/or reduction in amplitude.  2 Moderately impaired. Definite and early fatiguing. May have occasional arrests in movement.  3 Severely impaired. Frequent hesitation in initiating movements or arrests in ongoing movement.  4 Can barely perform the task. |
| ***AXIAL BRADYKINESIA*** | |
| ***1. Arising from chair***  0 Normal.  1 Slow or may need more than one attempt.  2 Pushes self up from arms of seat.  3 Tends to fall back and may have to try more than one time, but can get without help.  4 Unable to arise without help or cannot stand.  ***2. Sitting down***  0 Normal.  1 Slightly stiff or awkward.  2 Easily positions self before chair, but descent into chair is uncontrolled.  3 Has difficulty finding chair behind him/her and descent is uncontrolled.  4 Unable to test because of severe postural instability.  ***3. Posture***  0 Normal erect.  1 Not quite erect, slightly stooped posture; could be normal for an older person.  2 Moderately stooped posture, definitely abnormal; can be slightly leaning to one side.  3 Severely stooped posture with kyphosis; can be moderately leaning to one side.  4 Marked flexion with extreme abnormality of posture. | ***4. Gait***  0 Normal.  1 Walks slowly, may shuffle with short steps, but no festination or propulsion.  2 Walks with difficulty, but requires little or no assistance; may have some festinations, short steps, or propulsion.  3 Severe disturbance of gait, requiring assistance.  4 Cannot walk at all, even with assistance.  ***5. Postural stability***  0 Normal.  1 Retropulsion, but recovers unaided.  2 Absence of postural response; would fall if not caught by examiner  3 Very unstable, tends to lose balance spontaneously.  4 Unable to stand without assistance.  ***6. Body bradykinesia and hypokinesia***  0 None.  1 Minimal slowness, giving movement a deliberate character; could be normal for some persons. Possible reduced amplitude.  2 Mild degree of slowness and poverty of movement which is definitely abnormal. Alternatively, some reduced amplitude.  3 Moderate slowness, poverty or small amplitude of movements.  4 Marked slowness, poverty or small amplitude of movements |

**NNIPPS-PPS 5/6**

| ***OCULOR MOTOR FUNCTION*** | ***AXIAL DYSTONIA*** |
| --- | --- |
| ***1. Speed of voluntary upward saccades***  0 Normal speed.  1 Mild slowness.  2 Definite slowness.  3 Severe slowness or no saccades.  ***2. Amplitude of voluntary upward saccades***  0 Not hypometric.  1 Mild limitation.  2 Moderate limitation.  3 Severe limitation.  ***3. Speed of voluntary downward saccades***  0 Normal speed.  1 Mild slowness.  2 Definite slowness.  3 Severe slowness or no saccades.  ***4. Amplitude of voluntary downward saccades***  0 Not hypometric.  1 Mild limitation.  2 Moderate limitation.  3 Severe limitation.  ***5. Speed of voluntary left and right saccades***  0 *Normal speed.*  1 Mild slowness.  2. Definite slowness.  3 Severe slowness or no saccades.  ***6. Amplitude of voluntary left and right saccades***  0 Not hypometric.  1 Mild limitation.  2 Moderate limitation.  3 Severe limitation.  ***7.Eyelid dysfunction***  0 None.  1 Mild inhibition of opening or closing or mild blepharospasm : no visual disability.  2 Moderate lid-opening inhibition or blepharospasm causing partial visual disability.  3 Functional blindness or near-blindness because of involuntary eyelid closure. | ***1. Face***  0 Absent.  1 Subtle or present : only when activated by other movement.  2 Obvious but not continuous.  3 Only partial range of motion possible.  4 Continuous and disabling.  ***2. Neck***  0 *Absent*  1 Subtle or present : only when activated by other movement.  2 Obvious but not continuous  3 Only partial range of motion possible.  4 Continuous and disabling.  ***3. Trunk***  0 Absent.  1 Subtle or present : only when activated by other movement.  2 Obvious but not continuous.  3 Only partial range of motion possible.  4 Continuous and disabling. |
|  | ***LIMB DYSTONIA*** |
|  | ***1. Left Upper limb***  0 Absent.  1 Subtle or present : only when activated by other movement.  2 Obvious but not continuous.  3 Only partial range of motion possible.  4 Continuous and disabling.  ***2. Right Upper limb***  0 Absent.  1 Subtle or present : only when activated by other movement.  2 Obvious but not continuous.  3 Only partial range of motion possible.  4 Continuous and disabling.  ***3. Left Lower limb***  0 Absent.  1 Subtle or present : only when activated by other movement.  2 Obvious but not continuous.  3 Only partial range of motion possible.  4 Continuous and disabling.  ***4. Right lower limb***  0 Absent.  1 Subtle or present : only when activated by other movement.  2 Obvious but not continuous.  3 Only partial range of motion possible.  4 Continuous and disabling. |
| ***PYRAMIDAL SIGNS*** | |
| ***1. Masseter or jaw reflex***  0 Normal.  1 Exaggerated.  ***2. Babinski sign***  0 Negative (score the bilateral flexion).  1 Mute or equivocal (bilateral).  2 Extension (either side). | ***3. Deep tendon reflex Biceps, triceps, brachoradialis, quadriceps, triceps surae***  0 All Normal or any decreased.  1 Abnormally brisk (any abnormally brisk). |

**NNIPPS-PPS 6/6**

| ***CEREBELLAR FUNCTION*** | ***ORTHOSTATIC SYMPTOMS (interview)*** |
| --- | --- |
| ***1. Ataxia***  0 No abnormality.  1 Some abnormalities without disability.  2 Mild ataxia of one limb.  3 Moderate ataxia of trunk, walking or 2 limbs, or severe ataxia of one limb.  4 Moderate ataxia of 3 to 4 limbs, or severe ataxia of 2 limbs.  5 Severe ataxia of 3 to 4 limbs, or severe disability to coordinate movement related to ataxia.  ***2. Gaze-evoked nystagmus***  0 Normal.  1 Transient.  2 Persistent but moderate.  3 Persistent and severe.  *3****.* *Knee- tibia test : Lowering of heel in continuous axis***  **LEFT LEG**  0 Normal.  1 The movement is decomposed in several phases, without real jerks, or abnormally slow.  2 Lowering jerkily in the axis.  3 Lowering jerkily with lateral movements.  4 Lowering jerkily with extremely strong lateral movements.  9 Test impossible, specify why : __________________  **RIGHT LEG**  0 Normal.  1 The movement is decomposed in several phases, without real jerks, or abnormally slow.  2 Lowering jerkily in the axis.  3 Lowering jerkily with lateral movements.  4 Lowering jerkily with extremely strong lateral movements or tests impossible.  9 Test impossible, specify why : __________________  ***4. Finger to nose test (decomposition and dysmetria)***  **LEFT ARM**  0 No trouble.  1 Oscillating movement without decomposition of the movement.  2 Segmented movement in 2 phases and/or moderate dysmetria in reaching nose.  3 Segmented movement in more than 2 phases or considerable dysmetria in reaching nose.  4 Dysmetria preventing the patient from reaching nose.  9 Test impossible, specify why : __________________  **RIGHT ARM**  0 No trouble.  1 Oscillating movement without decomposition of the movement.  2 Segmented movement in 2 phases and/or moderate dysmetria in reaching nose.  3 Segmented movement in more than 2 phases or considerable dysmetria in reaching nose.  4 Dysmetria preventing the patient from reaching nose.  9 Test impossible, specify why : __________________ | ***1. In the past 12 months (6 months at follow-up): History of faintness or dizziness soon after standing-up from a sitting or lying position***  0 No  1 Yes  ***IF YES COMPLETE THE FOLLOWING (if no subsequent ratings = 0)***  ***1. How frequent did the patient get these symptoms ?***  1 Rarely, once a week or less.  1 Occasionally, several times a week.  3 Frequently, at least once a day.  4 Almost always, several times a day.  ***2. How does the patient rate the severity of these symptoms ?***  1 Mild.  2 Moderate.  3 Severe.  ***3. In the past 12 months (6 months at follow-up): how often did the patient end-up fainting soon after standing-up***  0 Never.  1 Once.  2 Twice.  3 Three times.  4 Four times.  5 Five or more times. |
|  | ***URINARY SYMPTOMS (interview)*** |
|  | ***1. In the past 12 months (6 months at follow-up): did the patient ever leak urine or lost cotrol of bladder function ?***  0 None or few drops less than once daily.  1 A few drops staining clothes daily.  2 Large amounts, but only when asleep, no pad required during day.  3 Occasional large amounts in daytime : pad required.  4 Consistent, requiring diaper or catheter awake and asleep.  ***2. In the past 12 months (6 months at follow-up): did the patient experience difficulties passing urine ?***  0 Never.  1 Occasionally.  2 Frequently.  3 Constantly or catheter in site.  ***3. In the past 12 months(6 months at follow-up): did the patient ever experience trouble completely emptying bladder ?***  0 Never.  1 Occasionally.  2 Frequently.  3 Constantly or catheter in site. |
