# Supplementary material for: Disease Severity and Progression in Progressive Supranuclear Palsy and Multiple System Atrophy: Validation of the NNIPPS – PARKINSON PLUS SCALE
Source: PLoS One. 2011 Aug 4;6(8):e22293. doi: 10.1371/journal.pone.0022293 (PMC3150329; doi:10.1371/journal.pone.0022293)
Supplement: Supporting information S2 — Details of psychometric validation methods and results. (DOC) [file pone.0022293.s002.doc]

**Supporting information S2**

**NNIPPS-PPS: details of psychometric validation methods and results**

The following psychometric properties were evaluated according to the recommendations of the -American Psychological Association [24].

*Face validity*: Raters of the trial were asked whether the items selected were clearly formulated, non ambiguous and relevant to measure severity of PSP and MSA. Comments on wording were collected and wording modified accordingly when relevant or Standard Operating Procedures clarified when necessary.

Content validity: A group of expert neurologists (n= 6) was asked if the items were representative of the main syndromes responsible for the severity of the diseases. Additions and/or deletion of item were performed as advised.

*Construct validity:*

No patient had more than 10 out of 84 (12%) missing item scoring values. Patients with 1 to 10 items missing (11% of cases) were excluded from this analysis. The overall analysis population included complete records of 675 patients (PSP n= 317; MSA n= 358).

*Factor analysis and internal consistency:*

To investigate the underlying structure of the scale, the inclusion data set of all complete records (N= 675) was submitted to principal component analysis, with Kaiser’s criterion (eigenvalue ≥ 1) as method of extraction followed by varimax rotation, to obtain independent dimensions. The internal consistency of the total and dimensional scales was assessed with Cronbach’s α coefficient.

For all patients, dimensional sub-scores were calculated by summing the items within each factor.

For patients with missing item scorings (n=85), these were imputed using the mean value obtained in their corresponding diagnostic strata. These sub-scores were used for subsequent analysis.

*Convergent validity* was evaluated using data from all patients included in the Intent to Treat population (N= 760). Correlations of the NNIPPS-PPS scores at inclusion (total and dimensional sub-scores) with other clinical measures considered *a priori* as related to severity, were calculated using Spearman rank coefficients: -Hoehn & Yahr staging (HYS [26]) and Schwab & England daily activities scale (SEADL [27]) used in Parkinson’s disease, the investigator’s Clinical Global Impression of disease severity (CGI-ds [25]), the Mini Mental State Examination (MMSE [28]) and the Frontal Assessment Battery (FAB [29]) for cognition evaluation, and visual analog scales (VAS) of severity (ranging from 0– not affected to 100- severely affected) corresponding to the main syndromes including akinesia-rigidity, dysautonomia, cerebellar, pyramidal, bulbar/pseudo-bulbar, and behavioral or cognitive dysfunction. A 4-point Clinical Global Impression – Dysautonomia score (CGI-dys; 0= none, 3= severe) was used to assess overall severity for dysautonomia. Correlations with quality of life scales were also analyzed including total PDQ-8 score [30] and 8 sub-scores of the SF-36 health survey questionnaire [31].

The *discriminant validity* of the scale in relation to disease severity was checked by comparing sub-groups defined according to the CGI-ds grades: (i) low severity group combining “Borderline” and “Mild” ratings, (ii) “Moderately ill”, (iii) “Markedly ill”, and (iv) high severity group combining “Severely /Extremely ill” ratings. Group means for total NNIPPS-PPS score and dimensional NNIPPS-PPS sub-scores were compared using one-way anova with test of trend for linearity. Furthermore, comparisons were made between the two groups defined by the extreme values (low and high severity) using Student’s t tests.

Comparison of PSP and MSA group means (Student’s t test) were done to check for the dimensions predicted to be more severe in one or other strata (e.g., dysautonomia for MSA, or oculomotor function for PSP).

*Predictive validity:*

The relation between scores at inclusion (i.e., total score and dimensional sub-scores) and survival was evaluated using univariate and multivariate Cox model [32]. Relative risks and confidence intervals (CI) 95% were determined.
