# Supplementary material for: Disease Severity and Progression in Progressive Supranuclear Palsy and Multiple System Atrophy: Validation of the NNIPPS – PARKINSON PLUS SCALE
Source: PLoS One. 2011 Aug 4;6(8):e22293. doi: 10.1371/journal.pone.0022293 (PMC3150329; doi:10.1371/journal.pone.0022293)
Supplement: Table S1 — Factor analysis of the NNIPPS-Parkinson Plus Scale. Data from 675 patients (317 PSP and 358 MSA) with fully completed scales at inclusion were submitted to Principal Component Analysis (PCA). The analysis included 84 out of the 85 items of the scale as one item, “erectile dysfunction”, could not be included due to poor completion rate. 85 patients (11%) had 1 to 10 item scores missing and were therefore excluded from analysis. Fifteen factors were extracted following varimax rotation. Loadings>0.30 of each item with corresponding factor are listed. Only one item, “sensory complaints”, did not correlate with any factor. For further analysis, and on clinical grounds, the first factor was split into 2 clinical dimensions: ADL/mobility based on interview items, and Axial bradykinesia based on motor examination; the 2 tremor factors were combined into a single dimension (Tremor). (DOC) [file pone.0022293.s003.doc]

***Table S1. Factor analysis of the NNIPPS-Parkinson Plus Scale.***

| **Clinical dimension** | **Loading** | **Clinical dimension** | **Loading** |
| --- | --- | --- | --- |
| **ADL/Mobility Axial Bradykinesia** | **FACTOR 1** | **Bulbar**/**Pseudobulbar Symptoms** | **FACTOR 6** |
| Gait | 0.82 | Speech/ motor examination | 0.75 |
| Arising from chair | 0.81 | Speech/ ADL | 0.74 |
| Walking | 0.80 | Salivation | 0.58 |
| Postural stability | 0.76 | Facial expression | 0.49 |
| Sitting down | 0.74 | Cough | 0.44 |
| Dressing | 0.73 | Swallowing | 0.40 |
| Hygiene | 0.70 | *% Variance* | *3.6* |
| Turning in bed | 0.64 | **Rigidity** | **FACTOR 7** |
| Posture | 0.60 | L-lower extremity rigidity | 0.75 |
| Cutting food | 0.58 | R-lower extremity rigidity | 0.73 |
| Body Bradykinesia | 0.53 | L-upper extremity rigidity | 0.65 |
| Freezing | 0.52 | R-upper extremity rigidity | 0.65 |
| Falling | 0.50 | Neck rigidity | 0.34 |
| Handwriting | 0.43 | *% Variance* | *3.3* |
| *% Variance* | *9.4* | **Tremor at rest** | **FACTOR 8** |
| **Limb Bradykinesia** | **FACTOR 2** | R-foot tremor at rest | 0.83 |
| R-hand movements | 0.78 | L-foot tremor at rest | 0.79 |
| R-Alternate hand movement | 0.78 | R-hand tremor at rest | 0.62 |
| L-Alternate hand movement | 0.77 | L-hand tremor at rest | 0.62 |
| R-finger taps | 0.75 | *% Variance* | *3.1* |
| L-hand movements | 0.74 | **Limb Dystonia** | **FACTOR 9** |
| L-finger taps | 0.72 | R-lower limb dystonia | 0.85 |
| R-leg agility | 0.67 | L-lower limb dystonia | 0.83 |
| L-leg agility | 0,66 | R-upper limb dystonia | 0.68 |
| *% Variance* | *7.4* | L-upper limb dystonia | 0.65 |
| **Oculomotor Function** | **FACTOR 3** | *% Variance* | *3.1* |
| Speed of downward saccade | 0.86 | **Postural Tremor** | **FACTOR 10** |
| Speed of upward saccade | 0.86 | L hand Postural tremor | 0.82 |
| Amplitude. of downward saccade | 0.86 | R hand Postural tremor | 0.82 |
| Amplitude. of upward saccade | 0.85 | Tremor/ADL | 0.59 |
| Speed of R/L saccade | 0.81 | Face tremor | 0.36 |
| Amplitude. of R/L saccade | 0.78 | *% Variance* | *3.0* |
| Eyelid dysfunction | 0.54 | **Orthostatic Symptoms** | **FACTOR 11** |
| *% Variance* | *6.9* | Severity orthostatic signs | 0.91 |
| **Cerebellar Function** | **FACTOR 4** | Frequency orthostatic signs | 0.88 |
| Left Knee-tibia test | 0.85 | Fainting frequency | 0.72 |
| Right Knee-tibia test | 0.85 | *% Variance* | *2.9* |
| Left Finger /nose test | 0.83 | **Myoclonus** | **FACTOR 12** |
| Right Finger /nose test | 0.82 | Myoclonus in action | 0.78 |
| Ataxia | 0.80 | Myoclonus with stimulation | 0.78 |
| Gaze-evoked nystagmus | 0.49 | Myoclonus at rest | 0.76 |
| *% Variance* | *5.2* | *% Variance* | *2.6* |
| **Mental** **Function** | **FACTOR 5** | **Axial Dystonia** | **FACTOR 13** |
| Loss of concentration | 0.74 | Neck dystonia | 0.78 |
| Motivation/Initiative | 0.68 | Trunk dystonia | 0.77 |
| Withdrawal | 0.67 | Face dystonia | 0.61 |
| Bradyphrenia | 0.66 | *% Variance* | *2.5* |
| Intellectual impairment | 0.65 | **Urinary Symptoms** | **FACTOR 14** |
| Grasping | 0.44 | Trouble emptying bladder | 0.80 |
| Depression | 0.40 | Difficulties passing urine | 0.78 |
| Aggressiveness | 0.38 | Loss control bladder function | 0.56 |
| Emotional Incontinence | 0.37 | *% Variance* | *2.3* |
| Thought disorder | 0.33 | **Pyramidal Symptoms** | **FACTOR 15** |
| *% Variance* | *4.2* | Deep tendon reflex | 0.67 |
|  |  | Babinski sign | 0.65 |
|  |  | Masseter reflex | 0.54 |
|  |  | *% Variance* | *2.1* |
|  |  | ***All dimensions total % Variance*** | ***61.6*** |
