# Supplementary material for: Disease Severity and Progression in Progressive Supranuclear Palsy and Multiple System Atrophy: Validation of the NNIPPS – PARKINSON PLUS SCALE
Source: PLoS One. 2011 Aug 4;6(8):e22293. doi: 10.1371/journal.pone.0022293 (PMC3150329; doi:10.1371/journal.pone.0022293)
Supplement: Table S2 — Convergent validity. Spearman rank correlations of the 15 dimensional and total scores of the NNIPPS Parkinson Plus scale at entry with other measures of clinical severity: the Clinical Global Impression Disease severity (CGI-ds), Hoehn & Yahr staging (HYS), Schwab & England scale (SEADL), Visual analog Scale (VAS) of severity of clinical syndromes including akineto-rigidity (AKIN.), dysautonomia (DYSAUT.), cerebellar (CEREB.), pyramidal (PYRAM.), bulbar/pseudobulbar (BULB.), cognitive (COG.) and behavioral (BEHAV.), and two measures of cognition, the Frontal Assessment Battery (FAB) and Mini Mental State Examination (MMSE). Moderate to high coefficients (≥0.40) are in bold characters. * Of note, Oculomotor, Axial dystonia, Limb dystonia, Myoclonus and Tremor had no reference measures. ADL = Activities of Daily Living (DOC) [file pone.0022293.s004.doc]

**Table S2: Convergent validity - Correlations of the NNIPPS-PPS with other clinical severity** measures

|  | **CGI-ds** | **SEADL** | **HYS** | **VAS-AKIN.** | **VAS-**  **DYSAUT.** | **VAS-CEREB.** | **VAS-PYRAM.** | **VAS-BULB.** | **VAS-COG.** | **VAS- BEHAV.** | **FAB** | **MMSE** |
| --- | --- | --- | --- | --- | --- | --- | --- | --- | --- | --- | --- | --- |
| **ADL/ Mobility** | **0.73** | **-0.85** | **0.80** | **0.57** | 0.09 | -0.02 | 0.10 | 0.39 | 0.24 | 0.21 | -0.27 | -0.30 |
| **Axial bradykinesia** | **0.71** | **-0.81** | **0.83** | **0.58** | 0.10 | 0.03 | 0.11 | 0.32 | 0.18 | 0.15 | -0.19 | -0.27 |
| **Limb bradykinesia** | **0.49** | **-0.56** | **0.51** | **0.59** | 0.14 | -0.04 | 0.07 | 0.24 | 0.13 | 0.08 | -0.16 | -0.24 |
| **Rigidity** | **0.40** | **-0.46** | **0.41** | **0.62** | 0.13 | -0.08 | 0.11 | 0.18 | 0.11 | 0.05 | -0.14 | -0.20 |
| **Bulbar/Pseudo-bulbar** | **0.59** | **-0.59** | **0.56** | **0.50** | 0.02 | -0.12 | 0.11 | **0.54** | 0.25 | 0.19 | -0.22 | -0.26 |
| **Orthostatic** | 0.10 | -0.03 | 0.04 | 0.02 | **0.52** | 0.13 | -0.03 | -0.05 | -0.21 | -0.17 | 0.17 | 0.09 |
| **Urinary** | 0.28 | -0.23 | 0.26 | 0.13 | **0.66** | 0.22 | 0.02 | -0.01 | -0.15 | -0.07 | 0.14 | 0.08 |
| **Pyramidal** | 0.08 | -0.09 | 0.09 | 0.12 | -0.01 | 0.05 | **0.74** | 0.17 | -0.01 | 0.04 | -0.02 | -0.08 |
| **Mental** | 0.25 | -0.35 | 0.23 | 0.16 | -0.20 | -0.17 | 0.02 | 0.31 | **0.61** | **0.54** | **-0.49** | **-0.46** |
| **Cerebellar** | 0.03 | -0.02 | 0.05 | -0.15 | 0.36 | **0.76** | 0.11 | -0.05 | -0.17 | -0.10 | 0.12 | 0.10 |
| ***Oculomotor** | 0.20 | -0.27 | 0.25 | 0.16 | **-0.51** | -0.33 | 0.04 | 0.36 | **0.45** | 0.33 | **-0.47** | -0.36 |
| ***Axial dystonia** | 0.16 | -0.11 | 0.13 | 0.16 | -0.02 | -0.07 | 0.12 | 0.08 | 0.14 | 0.09 | -0.13 | -0.09 |
| ***Limb dystonia** | 0.12 | -0.16 | 0.15 | 0.14 | -0.02 | -0.06 | 0.04 | 0.03 | 0.08 | 0.06 | -0.09 | 0.01 |
| ***Myoclonus** | 0.18 | -0.15 | 0.15 | 0.18 | 0.17 | 0.03 | 0.06 | 0.03 | -0.04 | -0.10 | 0.05 | -0.01 |
| ***Tremor** | 0.11 | -0.12 | 0.07 | 0.11 | 0.18 | 0.06 | -0.05 | -0.07 | -0.06 | -0.03 | 0.08 | -0.01 |
| **TOTAL SCORE** | **0.72** | **-0.80** | **0.76** | **0.61** | 0.15 | 0.004 | 0.14 | **0.42** | 0.30 | 0.25 | -0.33 | -0.35 |
