# Supplementary material for: Disease Severity and Progression in Progressive Supranuclear Palsy and Multiple System Atrophy: Validation of the NNIPPS – PARKINSON PLUS SCALE
Source: PLoS One. 2011 Aug 4;6(8):e22293. doi: 10.1371/journal.pone.0022293 (PMC3150329; doi:10.1371/journal.pone.0022293)
Supplement: Table S3 — Predictive Validity - Multivariate stepwise Cox model survival analysis. * Strata codes: 0 = PSP, 1 = MSA. Df = degrees of freedom, SD = standard deviation RR = relative risk, CI = confidence interval. Candidate covariates included strata, gender, disease duration, age at inclusion, age at onset of symptoms, Hoehn & Yahr Staging, Schwab & England Activities of Daily Living, Clinician Global Impression (CGI)-disease severity and Clinician Global Impression (CGI)-Dysautonomia score. The stepwise Cox model retained (by order of entry), the NNIPPS-PPS total score, CGI-dysautonomia, Disease duration, CGI-disease severity, and Strata as the best set of independent predictors. (DOC) [file pone.0022293.s005.doc]

**Table S3: Predictive Validity** - **Multivariate stepwise Cox model survival analysis.**

| Variables | Df | β (SD) | Khi2 | P>Khi2 (Wald) | RR [95%CI] |
| --- | --- | --- | --- | --- | --- |
| TOTAL SCORE PPS | 1 | 0,014 (0,002) | 32,00 | < 0,0001 | 1,014 [1,009-1,018] |
| CGI dysautonomia | 1 | 0,278 (0,085) | 10,64 | 0,0011 | 1,321 [1,117-1,561] |
| Disease Duration | 1 | -0,097 (0,030) | 10,38 | 0,0013 | 0,907 [0,855-0,963] |
| CGI disease severity | 1 | 0,206 (0,080) | 6,64 | 0,0100 | 1,229 [1,051-1,438] |
| STRATA* | 1 | -0,322 (0,151) | 4,56 | 0,0327 | 0,724 [0,539-0,974] |
